# Do People with Type 2 Diabetes Think They are Unhealthy? A Cross-Sectional Study in Celaya, Mexico

**DOI:** 10.5195/cajgh.2015.207

**Published:** 2015-05-28

**Authors:** Karla C. Paz-Salinas, Nicolas Padilla-Raygoza, Silvia C. Delgado-Sandoval, Georgina Olvera-Villanueva, Ma Laura Ruiz-Paloalto

**Affiliations:** 1 Division of Health Sciences and Engineering, Campus Celaya Salvatierra, University of Guanajuato, Mexico;; 2 Department of Nursing and Obstetrics, Campus Celaya Salvatierra, University of Guanajuato, Mexico;; 3 Department of Clinical Nursing, Campus Celaya Salvatierra, University of Guanajuato, Mexico

**Keywords:** Type 2 diabetes, lifestyle, overweight, obesity

## Abstract

**Background:**

Type 2 diabetes is a chronic disease that presents a significant burden on health care systems in many countries. With the rise of obesity, the incidence of Type 2 diabetes has also been steadily increasing. A healthy lifestyle and understanding of diabetes management are important factors for delaying the onset of comorbidities associated with Type 2 diabetes. The objective of this study was to evaluate the self-perception of health in individuals with Type 2 diabetes as it relates to BMI status, which has important implications for the implementation of preventive programs.

**Methods:**

A cross-sectional lifestyle survey was implemented in the region of Celaya, Guanajuato, Mexico, targeting 100 participants diagnosed with Type 2 diabetes. Anthropometric measurements and participant characteristics were also obtained. Fisher’s exact test was used to determine if the proportions of lifestyles perceptions differed by BMI status.

**Results:**

Participants had a mean age of 56.12 ± 10.26, a mean BMI of 29.13 ± 5.48 kg/m^2^, were mostly married (67.0%), and female (70.0%). None of the normal weight participants perceived themselves as unhealthy. 95% of overweight/obese participants perceived themselves to be healthy, despite a diagnosis of diabetes and being overweight/obese, while only 5% perceived themselves to be unhealthy. However, these differences in the perceptions of health classified by BMI status were not statistically significant (*p* = 0.42).

**Conclusion:**

Our findings indicate that overweight and obese persons with Type 2 diabetes in Celaya, Mexico may have misperceptions about their own health, even though these findings were not statistically significant. These preliminary data highlight the importance of implementing prevention and educational programs among those with diabetes, in order to combat health misperceptions and raise awareness about the dangers of diabetes and obesity. Furthermore, more research with larger sample sizes is needed in order to fully understand the effects of perception of health on actual health.

Type 2 diabetes is a chronic disease, which presents a significant burden on health services in many countries,^1^,^2^ including low income nations such as Afghanistan^3^ and Bangladesh,^4^ and middle income countries such as Kazakhstan^5^ and India.^6^ Type 2 diabetes is a metabolic disorder characterized by a deficit in the production or release of insulin, increasing glucose levels in plasma.^7^,^8^ This chronic disease and its complications are a major cause of morbidity and mortality in Mexico,^9^ with over 400,000 new cases reported in Mexico each year.^10^

Despite the benefits of maintaining a healthy lifestyle to control Type 2 diabetes, many diabetic patients do not make healthy lifestyle choices. For example, Mexico is now one of the most obese countries in the world with over 1 in 3 adults classified as obese.^11^ Lifestyle is the set of behaviors a person adopts in the maintenance of health or the occurrence/prevention of disease. Healthier lifestyles generally lead to better outcomes while unhealthier lifestyles lead to multiple diseases or disorders.^12^,^13^ Kickbusch et al. defined lifestyle as the general way of life based on the interaction of life conditions and individual behavior patterns, determined by social-cultural factors and an individual’s personal characteristics.^14^

Physical activity levels and dietary habits are related to body mass index (BMI), blood glucose levels, HDL cholesterol, and triglycerides, which are in turn related to Type 2 diabetes.^12^,^15^,^16^ It is essential to engage in regular physical activity and to carefully monitor food consumption in order to avoid diabetic complications. Lifestyle is a major contributor to disease and health outcomes, with a general understanding that if one wants to be a healthier person, have a healthier family, and live in a healthier community, one must make good lifestyle choices. However, since adverse effects of unhealthy lifestyles do not manifest immediately, people can fall into a vicious cycle of unhealthy behaviors and resist healthy lifestyle options due to the fact that they do not feel ill, causing a slow but progressive deterioration of health which gives rise to expensive chronic diseases.^13^,^15^,^16^

Self-perception of one’s health impacts the individual’s choice of lifestyle. Self-perception is the looking inward of oneself. Low concordance has been reported between nutritional status and self-perception of body image;^17^ for example, previous research in Europe demonstrated that 65% of men and 32% women underestimated their body weight.^18^ Lifestyle according to self-perception can be modified by improving knowledge of what is a desirable healthy lifestyle, and reversing misperceptions of self-health.

This study is of particular importance due to the high rates of obesity in Mexico,^11^ as self-perception of health can contribute to obesity, which in turn can exacerbate Type 2 diabetes. To our knowledge, no studies of this nature have been implemented in Mexico. The objective of this study was to determine if there is a relationship between the self-perception of lifestyle and BMI status in people with Type 2 diabetes residing in Celaya, Mexico.

## Methods

The study protocol was reviewed and approved by the Research Committee and the Bioethics Committee of the Division of Health Sciences and Engineering Campus Celaya Salvatierra, University of Guanajuato, Mexico. Participants were asked to provide written informed consent. Post-consent, participants completed a lifestyle questionnaire^19^ and anthropometric measurements were taken.

This study was a cross-sectional, community-based study on people with Type 2 diabetes registered in Mutual Help Groups (MHG) in the region of Celaya, Guanajuato. Participants were selected by simple random sampling. Inclusion criteria were the following: prior diagnosis of Type 2 diabetes, age of 18 years and older, male or female. Exclusion criteria were the following: individuals under 18 years of age and those without a diagnosis of Type 2 diabetes.

The lifestyle questionnaire provided a dichotomous variable: self-perception lifestyle (SPLS), which takes into consideration habits regarding physical activity, diet, smoking, alcohol consumption, self-care, accident prevention, moral values, environment, stress and social support, and sexuality.^19^ A healthy perception is categorized as 41–80 points, and an unhealthy perception is categorized as ≤40 points.^19^ The questionnaire measures self-perception of lifestyle but cannot measure actual health status. BMI status was categorized as a dichotomous variable. Normal BMI status was categorized as ≤25 kg/m^2^, and overweight/obese BMI status was categorized as >25 kg/m^2^.

### Sample size

Based on previous studies, sample size was calculated by assuming that 70% of those with an unhealthy lifestyle perception were overweight/obese and 40% of those with a healthy lifestyle were overweight/obese. The minimum required sample size was 42 for both groups (unhealthy lifestyle perception and healthy lifestyle perception), with 95% precision and 80% power (Epidat, 3.1, 2006, Xunta de Galicia and Pan American Health Organization).

### Statistical analysis

Descriptive statistics were obtained for basic participant characteristics. Fisher’s exact test was used to analyze the association of lifestyle perception and BMI status. All statistical tests were performed using STATA 13.0® (Stata Corp., College Station, TX, USA).

## Results

The 100 participants had a mean age of 56.12 ± 10.26 years (70% female, 67% married, mean BMI of 29.13 ± 5.48 kg/m^2^). Participant characteristics are summarized in Table 1.

81% of participants were classified as overweight/obese, and 96% of all participants perceived that they had a healthy lifestyle (Table 2). In the overweight/obese group, 5% perceived themselves as having an unhealthy lifestyle, while 95% perceived themselves as having a healthy lifestyle. This demonstrates that these people may have a distorted lifestyle perception; however, these findings were not statistically significant (*p* = 0.42).

## Discussion

The purpose of this study was to evaluate the self-perception of health in individuals with Type 2 diabetes. Only 4% of all participants were found to perceive themselves as having an unhealthy lifestyle; however, more were expected from the initial power analyses, which was a major disadvantage. One disadvantage was that the sample size was insufficient to detect statistically significant associations. Another disadvantage is that the sample was largely of a lower level of education, which has been found to be associated with obesity and negative health outcomes.^20^,^21^ In this particular sample, a large percentage of study participants was female. This could be due to the fact that females tend to utilize health services more than males; however, similar results were reported by Lopez-Carmona et al. in a diabetic lifestyle instrument validation study.^22^

Of the 81% classified as overweight/obese, only 5% reported having an unhealthy lifestyle perception, while 95% reported having a healthy lifestyle. This confirmed that there is a distorted perception of lifestyle, which can lead to exacerbation of diabetes symptoms.

In a Ugandan study, Mayega et al*.*, reported that only 14% of people with a high intake of fat perceived their diet as unhealthy.^23^ In Uganda, obesity is seen as “success” in non diabetic subjects and is desirable among women (“big is better”); weight loss is stigmatized as being sick and associated HIV / AIDS.^23^ In Denmark, Ulrichsen et al., reported that among 680 people with Type 2 diabetes, 36% were obese and 25% had a healthy diet.^24^ In Mexico, Chavez-Courtois, et al. studied a group of women with gestational diabetes and reported that women perceive physical activity and food as fundamental in controlling diabetes, although this does not mean that the measures on adequate physical activity and nutrition were implemented as part of treatment for diabetes.^25^

Patients with Type 2 diabetes typically perceived themselves as leading a healthy lifestyle; however, 81% of participants were overweight/obese, which is not desirable in diabetic patients due to the increased risk of diabetic complications or an earlier presentation of diabetic complications.

Patients with Type 2 diabetes receive a large amount of information concerning the benefits of a healthy lifestyle, and it is possible that this knowledge can generate an appropriate perception of a healthier lifestyle. This is especially important for those who are overweight or obese. We concluded that self-perception or self-reported lifestyle is not necessarily accurate and cannot be used in future research studies of this population without adding objective measures. Future research should focus on 1) the association of the self-perception of lifestyle and co-morbidities in diabetic patients, such as eye diseases, circulatory diseases, stroke, heart diseases, and 2) designing effective educational interventions to increase the awareness of unhealthy lifestyles among diabetic patients.

## Figures and Tables

**Table 1:** Qualitative socio-demographic characteristics of participants with Type 2 diabetes

Variables	Participants with Type 2 Diabetes N (%)
Gender	
Male	30 (30.0)
Female	70 (70.0)
Marital status	
Single	13 (13.0)
Married	67 (67.0)
Divorced	3 (3.0)
Separated	4 (4.0)
Widowed	9 (9.0)
Free union	4 (4.0)
Education	
None	28 (28.0)
Elementary (Grades 1–6)	27 (27.0)
Secondary (Grades 7–8)	19 (19.0)
High School (Grades 9–12)	12 (12.0)
Bachelor degree	12 (12.0)
Graduate degree	2 (2.0)

**Table 2:** Tabulation between perceived lifestyle and status of overweight and obesity in participants with Type 2 diabetes

Variables	Normal Weight N (%)	Overweight/Obese N (%)
Perceived lifestyle		
Unhealthy	0 (0.0)	4 (4.9)
Healthy	19 (100.0)	77 (95.1)

*Note*. Normal weight are those with a BMI < 25 kg/m^2^. Overweight/Obese are those with a BMI ≥ 25 kg/m^2^.
